# Characteristics of cultured desmoid cells with different CTNNB1 mutation status

**DOI:** 10.1002/cam4.582

**Published:** 2015-12-21

**Authors:** Shunsuke Hamada, Hiroshi Urakawa, Eiji Kozawa, Eisuke Arai, Kunihiro Ikuta, Tomohisa Sakai, Naoki Ishiguro, Yoshihiro Nishida

**Affiliations:** ^1^ Department of Orthopaedic SurgeryNagoya University Graduate School and School of MedicineNagoyaJapan

**Keywords:** *β*‐Catenin, CTNNB1 mutation, desmoid tumor, meloxicam, Wnt/*β*‐catenin pathway

## Abstract

Desmoid tumors are benign mesenchymal neoplasms with a locally aggressive nature. The mutational status of *β*‐catenin gene (CTNNB1) is presumed to affect the tumorous activity of the cells. In this study, we isolated three kinds of desmoid cell with different CTNNB1 status, and compared their characteristics. Cells were isolated from three patients with abdominal wall desmoid during surgery, all of which were resistant to meloxicam treatment. The mutational status of the CTNNB1 exon 3 was determined for both parental tumor tissues and isolated cultured cells. *β*‐catenin expression was determined with immunohistochemistry. Responsiveness to meloxicam was investigated with MTS assay together with COX‐2 immunostaining. mRNA expressions of downstream molecules of Wnt/*β*‐catenin pathway were determined with real‐time RT‐PCR. Three kinds of cell isolated from desmoid tumors harboring different CTNNB1 mutation status (wild type, T41A, and S45F), all exhibited a spindle shape. These isolated cells could be cultured until the 20th passage with unchanged proliferative activity. Nuclear accumulation of *β*‐catenin was observed in all cultured cells, particularly in those with S45F. Proliferating activity was significantly suppressed by meloxicam (25 *μ*mol/L, *P* < 0.007) in all three cell cultures, of which parental desmoid was resistant to meloxicam clinically. The mRNA expressions of Axin2, c‐Myc, and Cyclin D1 differently increased in the three cultured cell types as compared with those in human skin fibroblast cells (HDF). Inhibitors of Wnt/*β*‐catenin pathway downregulated Axin2, c‐Myc, and Cyclin D1 significantly. Isolated and cultured desmoid tumor cells harboring any one of the CTNNB1 mutation status had unique characteristics, and could be useful to investigate desmoid tumors with different mutation status of CTNNB1.

## Introduction

Desmoid‐type fibromatosis is a benign, but locally aggressive fibroblastic tumor. The biological features are enigmatic due to the markedly high recurrence rate after planned surgery (range 34–54%) 1, 2 and occasional spontaneous regression 3. For this reason, a nonsurgical approach, such as hormone therapy, nonsteroidal anti‐inflammatory drugs, or tyrosine kinase inhibitors, has been applied in recent years 4. Definitive treatment has not yet been established due to the small numbers of desmoid patients and the limited efficacy of previously reported drugs. Moreover, efficacy cannot be predicted in advance of the therapy, which has been the most pressing demand of patients and physicians.

In desmoid tumors, the nuclear accumulation of *β*‐catenin has shown diagnostic potential in differentiating desmoid tumors from other similar fibroblastic lesions 5. Its nuclear accumulation has been considered to be a trigger of desmoid tumors and its positivity denotes tumor aggressiveness 6, 7. A correlation between the nuclear positivity of *β*‐catenin and efficacy of conservative treatment has recently been reported 8. Thus, aberrant accumulation of *β*‐catenin, which causes activation of the Wnt pathway, is considered to play crucial roles in desmoid tumor biology.

This aberrant accumulation is commonly caused by mutations of the Wnt pathway‐related gene, particularly somatic mutations at exon 3 of CTNNB1 (*β*‐catenin gene) in sporadic extraperitoneal desmoid tumors 9, 10, 11. A minority have mutations of the adenomatous polyposis (APC) gene, which is associated with familial adenomatous polyposis (FAP). CTNNB1 mutations of desmoid tumors generally occur at codon 41 or 45, with p.T41A (threonine to alanine), p.S45F (serine to phenylalanine), and p.S45P (serine to proline) being the most frequent 9, 10, 11. Recent studies have suggested that desmoid tumors with different CTNNB1 mutations have diverse tumorigenic potency against various treatment modalities. Desmoid tumors with S45F mutation had higher rates of local recurrence after surgery 10, 12, 13, and greater resistance to meloxicam treatment 14, whereas the efficacy of low‐dose chemotherapy was not associated with the mutation status of CTNNB1 15. Taking these findings into consideration, the mutation status of CTNNB1 in desmoid tumors would appear to alter not only tumorigenicity, but also the responsiveness to surgical and conservative treatment. However, the mechanism whereby the mutation status affects biological behavior has not been extensively investigated. This prompted us to isolate, culture, and characterize desmoid cells harboring different mutation status of CTNNB1 (wild type, S45F, and T41A). To exclude other possible factors affecting the biological behavior of desmoid cells, we selected these three cell types from tumors resistant to meloxicam treatment located in the abdominal wall of three young female patients.

## Material and Methods

### Tissue acquisition

Desmoid tissues were collected, and subjected to various experiments including cell culture and CTNNB1 mutation analysis. The experimental protocol was approved by the institutional review board of Nagoya University. Among patients prospectively treated with meloxicam 16, 17, 18, three young females with abdominal wall desmoids resistant to meloxicam (progressive disease according to Response Evaluation Criteria in Solid Tumors) were treated surgically. Part of the resected tumors, from which normal tissues were carefully removed, was applied for cell culture. The patients' ages at the time of surgery were 20, 30, and 39 years. All three resected tumors were histologically diagnosed as desmoid tumors by specialized pathologists including immunohistochemical analysis of *β*‐catenin.

### Cell cultures

The samples were cut into small pieces with a sterile scalpel, and were dissociated with 0.2 mg/mL proteinase in Dulbecco's modified Eagle's medium (DMEM) at 37°C for 3 h. The resulting cells were seeded into T‐75 flasks, and cultured in DMEM containing 10% fetal bovine serum (FBS), 100 U/mL penicillin, and 100 mg/mL streptomycin at 37°C in humidified atmosphere air plus 5% CO^2^. The medium was changed every 3–4 days. After the cells were grown to near confluency (passage #1), they were trypsinized and divided for continued in vitro culture. Successive experiments were performed with cell cultures of passage 5–15. Human skin fibroblasts (HDF; Detroit 551: ATCC, CCL‐110) were cultured in monolayers and used as control cells.

### Mutation analysis of CTNNB1

DNA of tumors was extracted from both 5‐*μ*m thick formalin‐fixed, paraffin‐embedded specimens and cell lysate of monolayer cultures, using High Pure Polymerase Chain Reaction (PCR) Template Preparation Kit (Roche, Basel, Switzerland), according to the manufacturer's instructions. Quality of DNA was confirmed with A_260_/A_280_ ratio; more than 1.8. Extracted DNA was amplified by PCR with 40 cycles at an annealing temperature of 58°C with specific primer pairs for exon 3 of CTNNB1 using the LightCycler 480 System (Roche). We designed specific 2 primer pairs: forward 5′‐GATTTGATGGAGTTGGACATGG‐3′, reverse 5′‐TCTTCCTCAGGATTGCCTT‐3′, and forward 5′‐TGGAACCAGACAGAAAAGCG‐3′, reverse 5′‐TCAGGATTGCCTTTACCACTC ‐3′ (The expected sizes of amplified products were of 149 and 118 bp, respectively). The PCR products were segregated by 2% agarose gel electrophoresis, and gel bands of predicted size were extracted and purified using the QIAquick gel extraction kit (Qiagen, Valencia, CA). The purified products were subjected to direct sequencing using the above described forward primers with Applied Biosystems Big Dye Terminator V3.1 and Applied Biosystems 3730x DNA analyzer (Applied Biosystems, Foster City, CA) at FASMAC Co. Ltd. (Kanagawa, Japan). All sequencing results obtained with two different primer pairs were compared and confirmed as identical. Mutation site was determined with the databases of NCBI‐BLAST.

### Immunofluorescence staining

Immunofluorescence staining was performed to evaluate the nuclear accumulation of *β*‐catenin. The cells were grown to 40–60% confluency on sterile glass coverslips, fixed with 4% paraformaldehyde, washed once with phosphate buffered saline PBS, and soaked with 3% bovine serum albumin (BSA) for 30 min for blocking. Then, slides were incubated for 1 h at 37°C with anti‐*β*‐catenin rabbit polyclonal antibody (ab47426; Abcam, Cambridge, CA; 1:200 dilution) or anti‐COX‐2 goat polyclonal antibody (sc‐1747; Santa Cruz Biotechnology, Santa Cruz, CA; 1:500 dilution). After rinsing with PBS, fluorescent goat polyclonal anti‐rabbit IgG–H&L (Alexa Flour^®^ 488) (ab150077; Abcam; 1:1000 dilution) or donkey polyclonal anti‐goat IgG–H&L (Alexa Flour^®^ 488) (ab150129; Abcam; 1:1000 dilution) were used as secondary antibodies. After nuclear staining with DAPI(D1306; Life Technologies, Carlsbad, California), slides were analyzed under a fluorescence microscope. Nonimmune rabbit serum was substituted for the primary antibody as a negative control.

### Cell proliferation and apoptosis

Doubling time of the three cultured cell types was determined by cell growth assay. Desmoid tumor cells were seeded in 96‐well plates at 5 × 10^3^/well in medium supplemented with 10% FBS and allowed to adhere for 12 h. The subconfluent cells were exposed to 10% FBS medium with and without dimethyl sulfoxide DMSO containing 0–50 *μ*mol/L meloxicam. After treatment for 48 h, cell proliferation was measured by 3‐(4,5‐dimethylthiazol‐2‐yl)‐5‐(3‐carboxymethoxyphenyl)‐2‐(4‐sulfophenyl)‐2H‐tetrazolium, inner salt (MTS) assay using a CellTiter 96^®^ AQueous One Solution Cell Proliferation Assay (Promega, Fitchburg, WI). Absorbance intensity was determined on a microplate reader, Rainbow RC (Tecan Japan, Kawasaki, Japan) at 490 nm. Subconfluent cells were also exposed to 10% FBS medium with and without DMSO containing 25 *μ*mol/L meloxicam or 50 *μ*mol/L actinomycin‐D for 24 h, and apoptotic activity was evaluated by Caspase‐Glo^®^ 3/7 Assays (Promega) according to the manufacturer's instructions. Luminescent intensity was determined on a microplate reader, PowerScan4 (DS Parma Biomedical, Osaka, Japan).

### Real‐time RT‐PCR

The mRNA expression of target genes, Axin2, Cyclin D1, and c‐Myc in the Wnt/*β*‐catenin signaling pathway was determined by real‐time RT‐PCR. Total cellular RNA was isolated from cultured cells in monolayer using RNeasy Mini Kit (Qiagen), according to the manufacturer's instructions. Reverse transcribed cDNA was subjected to real‐time RT‐PCR for semiquantification of Axin2, Cyclin D1, and c‐Myc mRNAs using a LightCycler (Roche Diagnostics, Mannheim, Germany). The relative levels of these mRNA in a sample were expressed after normalization with GAPDH mRNA. The Axin2, Cyclin D1, c‐Myc, and GAPDH primer pairs were as follows: Axin2 sense; 5′‐TGTCTTAAAGGTCTTGAGGGTTGAC ‐3′, antisense; 5′‐CAACAGATCATCCCATCCAACA ‐3′ (predicted PCR product of 80 bp); Cyclin D1 sense; 5′‐CAGCTCCTGTGCTGCGAAG‐3′, antisense; 5′‐ACGGCAGGACCTCCTTCTG ‐3′ (predicted PCR product of 157 bp), c‐Myc sense; 5′‐TCTGGATCACCTTCTGCTGG ‐3′, antisense; 5′‐AGGATAGTCCTTCCGAGTGG ‐3′ (predicted PCR product of 126 bp), GAPDH sense 5′‐AGGTCGGAGTCAACGGATTTG‐3′, antisense, 5′‐TGTAAACCATGTAGTTGAGGTCA‐3′ (predicted PCR product of 123 bp).

### Effects of inhibitors for Wnt/*β*‐catenin pathway on mRNA expression of target molecules

To determine the effects of inhibitors for Wnt/*β*‐catenin pathway on gene expression of Axin2, Cyclin D1, and c‐Myc, two inhibitors (IWR‐1; Santa Cruz Biotechnology, Santa Cruz, Quercetin; Wako, Osaka, Japan) were used for cell cultures harboring CTNNB1 mutations. IWR‐1 (10 *μ*mol/L), which inhibits Wnt‐induced *β*‐catenin accumulation through stabilization of the destruction complex member Axin1 19, and Quercetin (20 *μ*mol/L), which blocks the *β*‐catenin–TCF (T‐cell factor)/lef (lymphoid enhancer factor)‐1 pathway 20, were added to desmoid cell culture in 60‐mm tissue cultured dishes (93060; TPP, Trasadingen, Switzerland) (2.0 × 10_4_ cells/cm^2^) for 24 h at 37°C. Cultured cells were subjected to total RNA purification and subsequent real‐time RT‐PCR to determine the effects on mRNA expression of Axin2, Cyclin D1, and c‐Myc.

### Statistical evaluation

All the in vitro quantitative experiments were performed three times, and analysis of variance followed by Bonferroni–Dunn post‐hoc test was used to assess differences between the means. The results are expressed throughout as the mean ± SD. All statistical analyses were performed using SPSS statistics 20 (IBM Corp. Armonk, NY). *P* < 0.05 was considered significant.

## Results

### Three cultured cell types with different CTNNB1 mutation status

**Figure 1:**
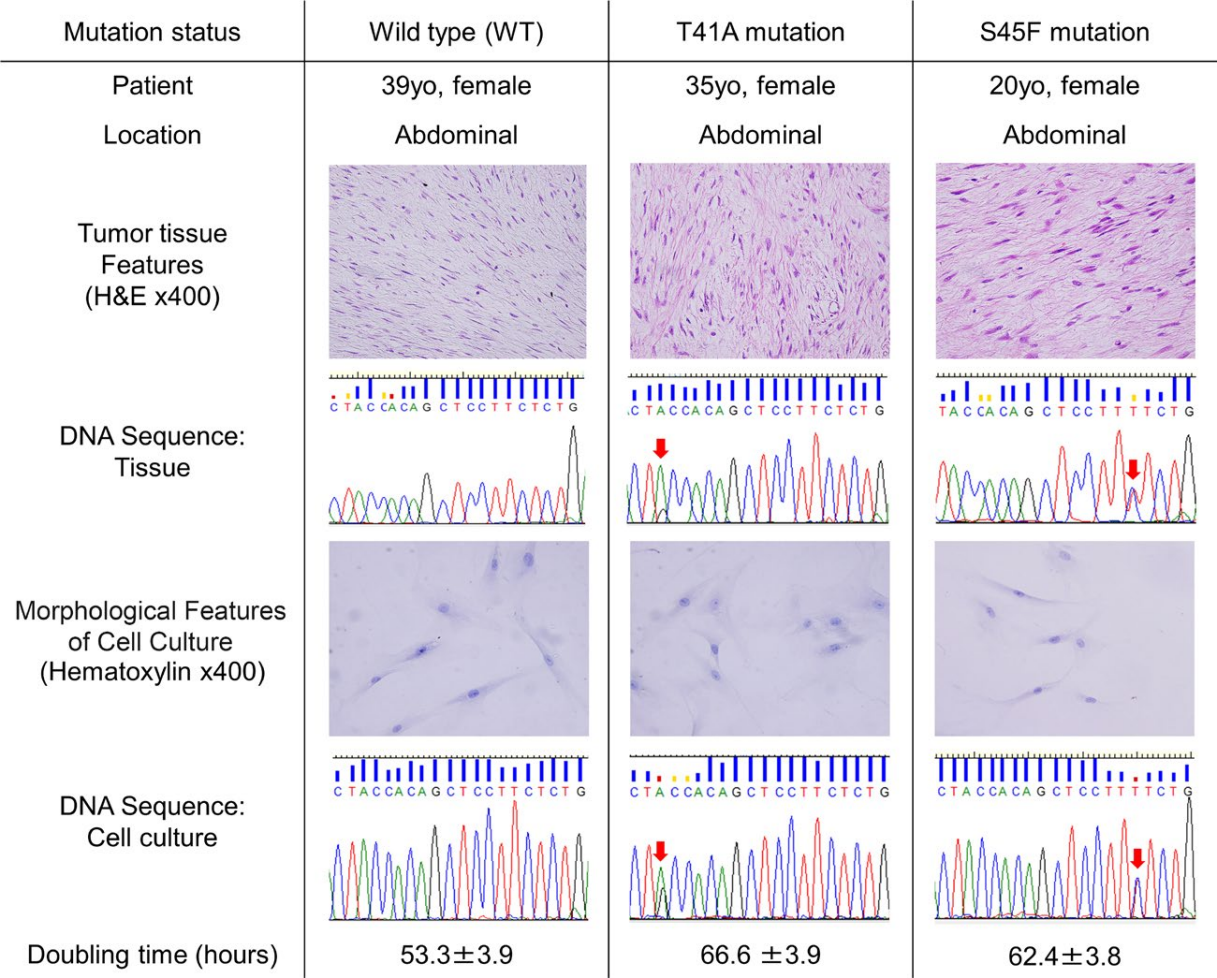
Parental desmoid tissues and isolated cell cultures. Histological and cell morphological features of three different CTNNB1 mutation status. Waveform data of DNA sequence at CTNNB1 exon3 and doubling time of each cell types are shown.

All three cultured cell types of desmoid tumors exhibited spindle‐shape, homogeneous fibroblast‐like morphology. Doubling time of cultured cells with T41A, S45F, wild type (WT) was 66.6, 62.4, and 53.3 h, respectively (Fig. 1). The three cultured cell types did not become senescent, and the growth behavior remained constant until the 20th passage.

Mutation analyses of CTNNB1 revealed that parental desmoid tumors harbored (WT), T41A, and S45F, which were identical with the respective mutation of the isolated cultured cells (Fig. 1).

### Expression of **β**‐catenin and COX‐2

**Figure 2:**
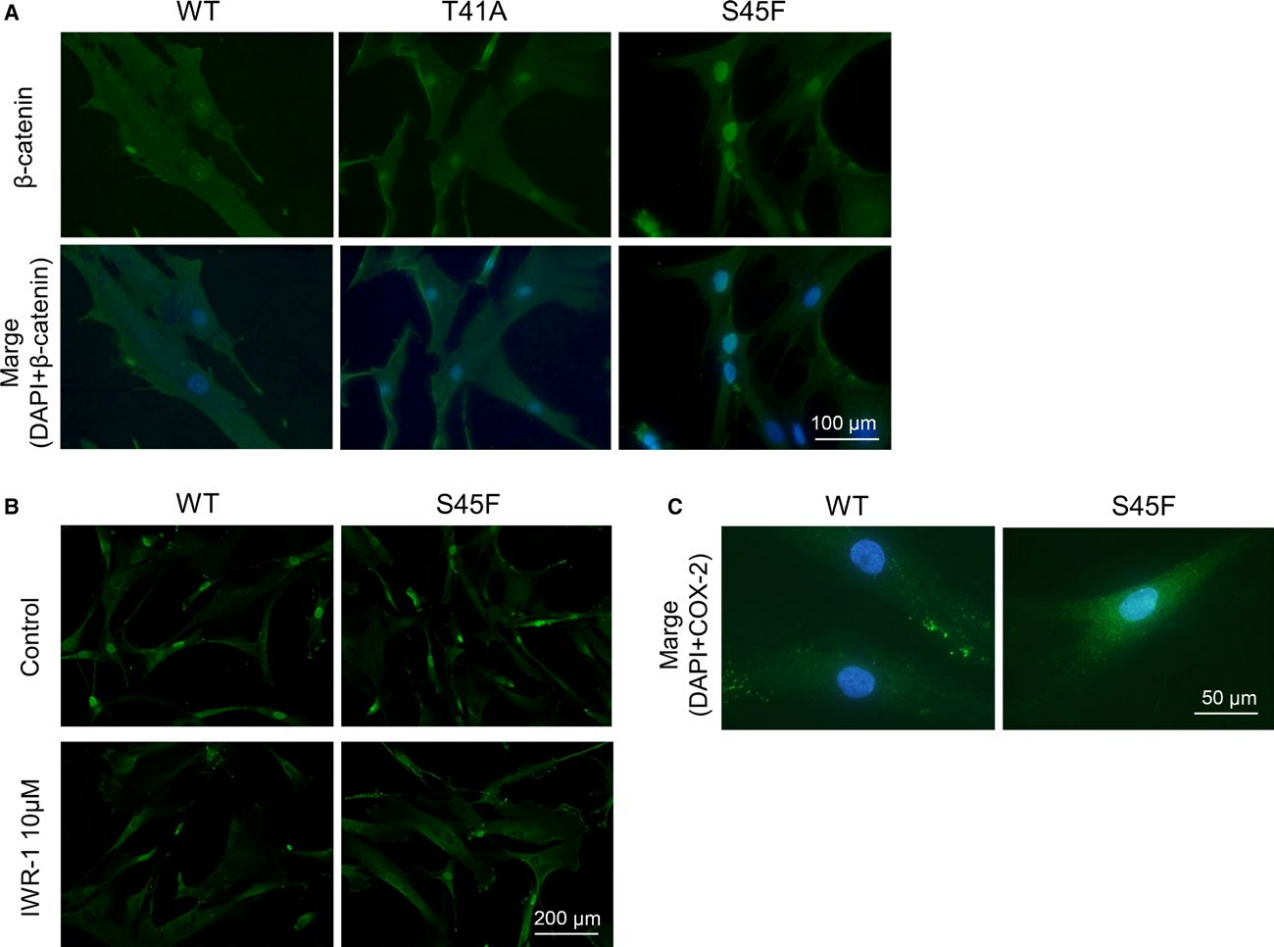
Immunofluorescence of *β*‐catenin and COX‐2 in desmoid cell cultures. (A) Immunofluorescence of *β*‐catenin (green) in desmoid cell cultures harboring each CTNNB1 mutation status. The DAPI (blue) staining of cell nuclei were merged with *β*‐catenin staining (original magnification, ×200). (B) Immunofluorescence of *β*‐catenin (green) with 10 *μ*mol/L of IWR‐1 (original magnification, ×100). (C) Fluorescent double staining for COX2 (green) and DAPI (Blue) (original magnification, ×400).

S45F harboring cells exhibited strong nuclear *β*‐catenin positivity. T41A and WT cultured cells showed intermediate and relatively weak staining (Fig. 2A). IWR‐1 treatment could not effectively downregulate nuclear *β*‐catenin accumulation (Fig. 2B). Immunofluorescent study for COX‐2 indicated strong positivity in cytoplasm, particularly around the nucleus, of S45F cells as compared with that of WT cells (Fig. 2C).

### Effects of meloxicam on cell proliferation and apoptosis

**Figure 3:**
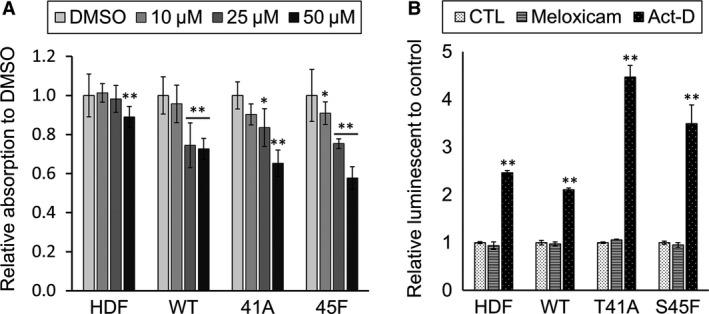
Effects of meloxicam on cell proliferation and apoptotic activity. (A) Cell proliferation of each cell culture was in a dose‐dependent manner with meloxicam at 0–50 *μ*mol/L for 48 h with MTS assay kit. (B) Effects of meloxicam on apoptosis in human skin fibroblast cells (HDF) and desmoid cell cultures. Caspase3/7 assay was performed with 25 *μ*mol/L meloxicam or 50 *μ*mol/L actinomycin‐D (Act‐D), relative luminescent to control (CTL) was exhibited. Bars show one standard deviation (SD) (*P < 0.05, **P < 0.01).

Inhibitory effects of meloxicam were evaluated in three cultured cell types, which were derived from desmoid tumors resistant to meloxicam clinically. Cell proliferation was inhibited in a dose‐dependent manner in all of them. It was significantly suppressed with 25 *μ*mol/L meloxicam in all three cell cultures (suppression rate ranging from 16–24%), whereas HDF was not affected (Fig. 3A). Higher dose (50 *μ*mol/L) inhibited HDF proliferation.

Actinomycin‐D (50 *μ*mol/L) significantly increased apoptotic activity in HDF, WT, T41A, and S45F cultured cells (*P* < 0.01). However, meloxicam did not induce apoptosis in any of the cultured cells (Fig. 3B).

### Steady state mRNA expression of Axin2/Cyclin D1/c‐Myc in three desmoid cell cultures

**Figure 4:**
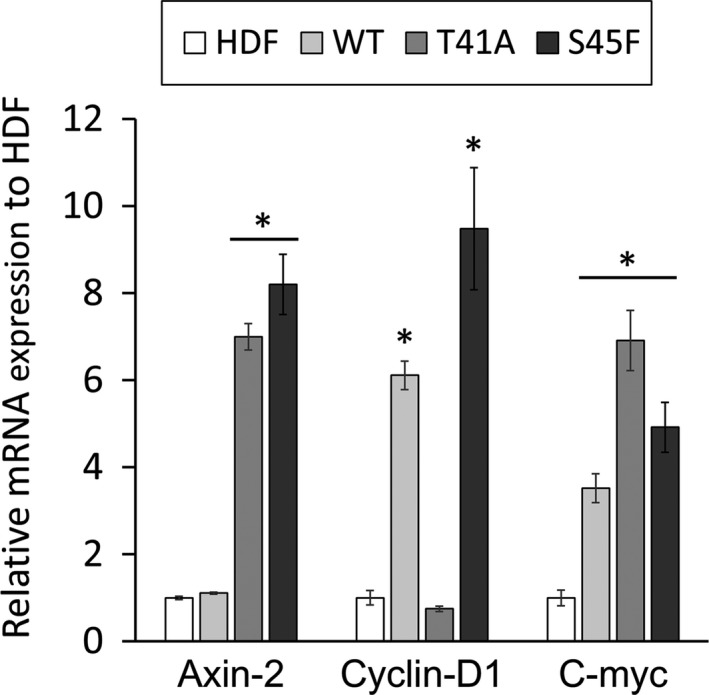
mRNA expression of the target genes of Wnt/*β*‐catenin in cultured cells from desmoid tumors. Expression level is depicted as *n*‐fold of the normalized amount of mRNA of human skin fibroblast cells HDF control cells. Bars show one standard deviation (SD) (*P < 0.01).

The mRNA expression of Axin2, Cyclin D1, and c‐Myc was significantly increased in the S45F‐mutated cells (*P* < 0.001, 8.2‐fold, 9.5‐fold, and 4.9‐fold, respectively) compared with that of HDF cells. mRNA of Axin2 and c‐Myc was increased in cultures with T41A mutation, and that of cyclin D1 and c‐Myc was in cultures with WT (*P *<* *0.001) (Fig. 4).

### Effects of inhibitors for Wnt/β‐catenin pathway on mRNA expression of Axin2/cyclin D1/c‐Myc

**Figure 5:**
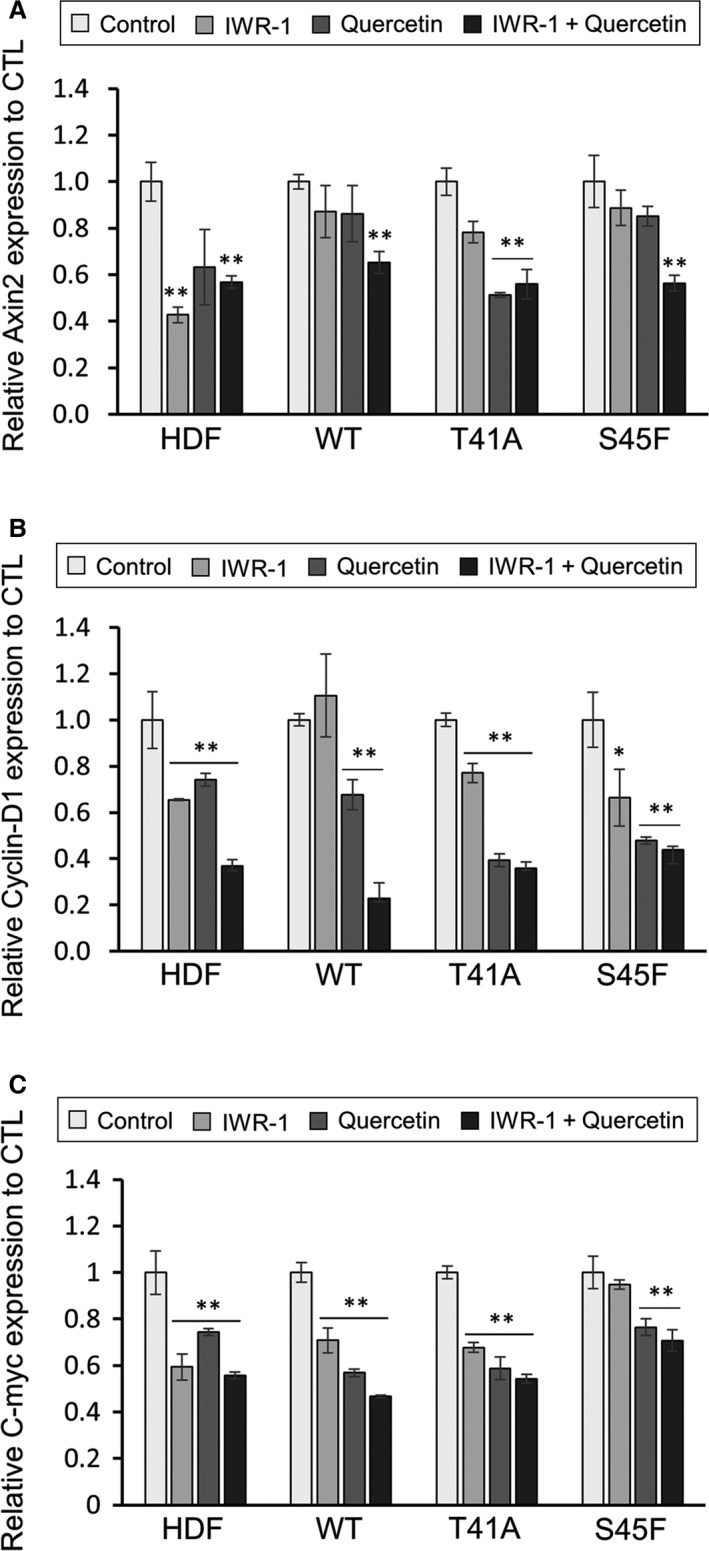
Effects of Wnt/*β*‐catenin inhibitors on mRNA expression of the target genes of Wnt/*β*‐catenin pathway. (A) Axin2. (B) CyclinD1. (C) C‐myc. IWR‐1 (10 *μ*mol/L) and quercetin (20 *μ*mol/L) were added to each cell culture. Expression level is depicted as *n*‐fold of the normalized mRNA of control cells. Bars show one standard deviation (SD) (*P < 0.05, **P < 0.01).

IWR‐1, which stabilizes Axin protein and subsequently promotes *β*‐catenin degradation, reduced to some extent the mRNA expression of Axin2, cyclin D1 and c‐Myc, whereas quercetin, which blocks the *β*‐catenin–TCF/Lef‐1 pathway, more markedly decreased the mRNA expression. The combined use of IWR‐1 and quercetin exerted additive inhibitory effects on mRNA expressions (Fig. 5A–C).

## Discussion

We successfully cultured and characterized desmoid cells harboring three different CTNNB1 mutation status: wild type, T41A, and S45F. In malignant tumors, mutation of CTNNB1 has been reported to locate between codon 32 and 45, the site of phosphorylation by GSK3*β* or CK1*α* 21, 22. It spans a much wider range compared with that in desmoid tumors, possibly explaining the paucity of reports describing correlations between specific mutation status and biological behavior in malignant tumors. On the other hand, several studies have demonstrated the relationship between mutation status of CTNNB1 and treatment outcome including surgery 10, 12, 13 and conservative therapy 14, 15 in patients with desmoid tumors. To obtain proof of this concept using in vitro study and investigate cell behavior with different molecular features, and to establish and characterize cell cultures with diverse mutation status of CTNNB1 will be important.

Cultured cells had homogenous fibroblast‐like features despite the different mutation status of CTNNB1. Although positivity of nuclear *β*‐catenin staining was strong in cells with S45F, doubling time was shortest in WT cells compared with those with T41A and S45F. On the other hand, downstream target gene of Wnt/*β*‐catenin signaling pathway including Axin2, Cyclin D1, and c‐Myc were upregulated in S45F cells compared with T41A and WT cells. Considering the results of previous reports that patients with S45F exhibited resistance to surgical and conservative treatment 10, 12, 13, 14, cells with S45F may exhibit the most aggressive biological behavior. Results of this study could not completely explain the in vivo behavior of desmoid cells. Part of the biological behavior will be affected by the environmental conditions in vivo, because patients with young age and/or extremity desmoid tumors had significant worse surgical outcome regardless of the mutation status 23, suggesting that host conditions may influence the tumorigenicity.

Based on a previous study in which COX‐2 blockade decreased cell proliferation of desmoid tumor in vitro, and inhibited the growth of desmoid tumors in a mouse model 24, a COX‐2 inhibitor, meloxicam, has been prospectively used for patients with extraperitoneal desmoid tumors in our institution 16, 18. Cultured cells in this study were all derived from tumors exhibiting resistance to meloxicam treatment clinically, suggesting that responsiveness to meloxicam may not be different in in vitro experiments among the three cultured cell cultures. Although positivity of COX‐2 immunofluorescence staining was stronger in S45F cells compared to other cells, responsiveness to meloxicam treatment was similar among cells. It will be explained by our previous report describing that responsiveness to meloxicam is not correlated with the COX‐2 stainability 8. Despite deriving from tumors resistant to meloxicam, the reason why meloxicam showed inhibitory effects in vitro of three cells may partly be due to the difference between in vitro and in vivo environment. On the other hand, apoptotic activity of actinomycin‐D was well characterized in the analyzed cell cultures. A previous study evaluated the proliferation rate of desmoid cell cultures with three COX blocking agents, sulindac, indomethacin, and 5,5‐Dimethyl‐3‐(3 fluorophenyl)‐4‐(4 methylsulphonal) phenyl‐2 (5H)‐furanone (DFU), and determined the concentration effective to inhibit the cell viability, although CTNNB1 mutation status was not considered in their study 24.

Nuclear accumulation of *β*‐catenin is considered to activate T‐cell factor, which in turn stimulates Tcf/Lef transcriptional gene expression 25, 26. The expression of these genes including Axin2, c‐Myc, Cyclin D1 has been shown to be increased in desmoid tumor 27, 28, 29. In this study, expression patterns of these target genes altered among cells harboring different mutation types. To determine these characteristics may help to understand the biological features of desmoid cells with different mutation type, and moreover, to evaluate the responsiveness of cells to drugs based on mutation type. Effectiveness of drugs had better be analyzed by not only cell viability test, but also gene expression patterns of target genes because downstream of Tcf/Lef transcription pathway should have crucial roles in tumorigenesis of desmoid tumors. Meneghello et al. reported results inconsistent with those of our study, namely that mRNA expression of cyclin D was decreased in all desmoid cells harboring different mutation status compared with control cells, whereas that of Axin2 increased 28. This discrepancy between our studies might be due to differences in the control cells used. The control cells used in their study showed the greatest proliferation as compared with desmoid cells. Another reason might be the heterogeneous origin of desmoid cells in their study, including male and female patients, age ranging from 31 to 53 years, and location in abdominal wall versus extremity. In contrast, the cells used in this study were all obtained from female patients, located on the abdominal wall, and with age ranging from 20 to 39, and thus seemingly had similar background features except for mutation status. The expression of all target genes in cells with S45F mutation, and two genes in those with WT or T41A mutation were increased compared with that in control cells. Generally, phosphorylation first occurs at S45 by CK1*α*, and subsequently at T41, S37, and S33 by GSK3*β* 30. Phosphorylation of S45 seems to be an essential process for the degradation of *β*‐catenin, suggesting S45F mutation, which may block the entire phosphorylation, and cause the most prominent accumulation of *β*‐catenin.

Different effects were observed of inhibitors (IWR‐1 and quecertin) for Wnt/beta‐catenin pathway on desmoid cells. Mutation of *β*‐catenin seems to be insulated from degradation by Axin/APC complex. IWR‐1, which stabilizes Axin protein and stimulates *β*‐catenin degradation might have less marked effects on TCF/Lef‐1, downstream of the Wnt/beta‐catenin pathway compared with quercetin, which blocks the TCF/Lef‐1 pathway. Present study using inhibitors of Wnt/beta‐catenin pathway revealed that desmoid cells harboring different mutation status had partly different response to these inhibitors. Although in vivo conditions should be taken into account in the future, investigating gene expression profile after treatment of various inhibitors may provide meaningful information.

There are several limitations in this study. First, although isolated desmoid cells were all obtained from females and from the abdominal wall with different mutation status, cell characteristics should be determined from different locations including extremity and neck regions where tumors occasionally show resistance to therapies. Another is that the behaviors of desmoid tumors are considerably affected by the host environment including age, gender, and location. Experiments using isolated cells in vitro could not easily mimic in vivo phenomena.

In conclusion, we successfully cultured and characterized three desmoid cell types with different CTNNB1 mutation status. Investigations using these cells will help to clarify the altered responsiveness of cells with different mutations to various therapies.

## Conflict of Interest

None declared.
